# Evaluation validation of a qPCR curve analysis method and conventional approaches

**DOI:** 10.1186/s12864-021-07986-4

**Published:** 2021-11-16

**Authors:** Yashu Zhang, Hongping Li, Shucheng Shang, Shuoyu Meng, Ting Lin, Yanhui Zhang, Haixing Liu

**Affiliations:** 1 Department of Information Science and Engineering, Ocean University of China, Qingdao, China; 2 Apexbio Biotechnology (Suzhou) Co., Ltd, Suzhou, China; 3 First Institute of Oceanography, Ministry of Natural Resources, Qingdao, China

**Keywords:** Reverse transcription quantitative polymerase chain reaction, Curve analysis method, C_q_MAN, Performance indicators

## Abstract

**Background:**

Reverse Transcription quantitative polymerase chain reaction (RT-qPCR) is a sensitive and reliable method for mRNA quantification and rapid analysis of gene expression from a large number of starting templates. It is based on the statistical significance of the beginning of exponential phase in real-time PCR kinetics, reflecting quantitative cycle of the initial target quantity and the efficiency of the PCR reaction (the fold increase of product per cycle).

**Results:**

We used the large clinical biomarker dataset and 94-replicates-4-dilutions set which was published previously as research tools, then proposed a new qPCR curve analysis method——C_q_MAN, to determine the position of quantitative cycle as well as the efficiency of the PCR reaction and applied in the calculations. To verify algorithm performance, 20 genes from biomarker and partial data with concentration gradients from 94-replicates-4-dilutions set of MYCN gene were used to compare our method with various publicly available methods and established a suitable evaluation index system.

**Conclusions:**

The results show that C_q_MAN method is comparable to other methods and can be a feasible method which applied to our self-developed qPCR data processing and analysis software, providing a simple tool for qPCR analysis.

**Supplementary Information:**

The online version contains supplementary material available at 10.1186/s12864-021-07986-4.

## Background

**Fig. 1:**
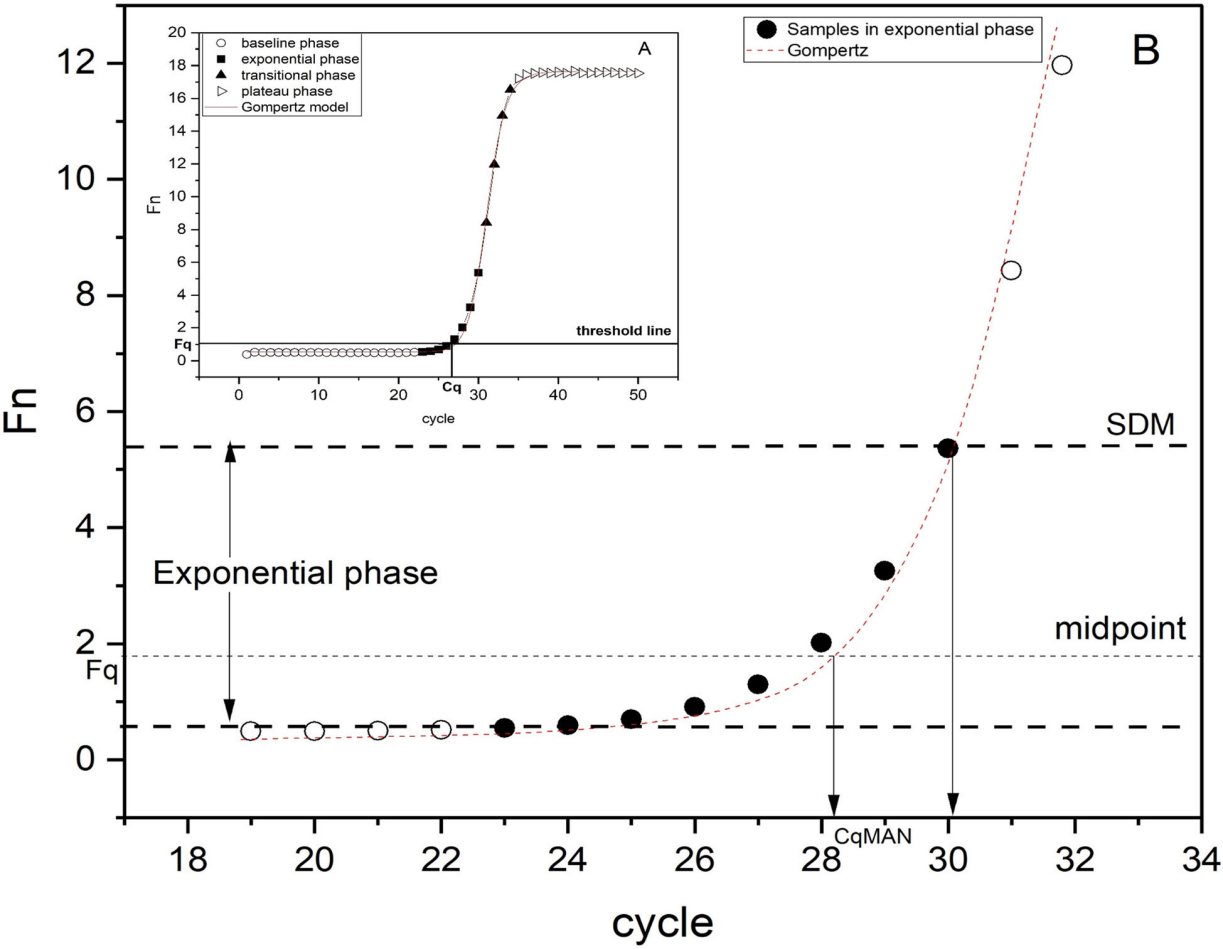
Amplification process and determination of parameter in C_q_MAN method. (OriginPro 2020b https://www.originlab.com/)

The working principle of the qPCR is to add fluorophore into the qPCR system, and use the fluorescence signal accumulation to detect the whole qPCR process [1]. The accumulated amount of DNA reaction products after fluorescent labeling is used as amplification data (expressed as amplification curves) can be used to determine the initial target quantity (called N_0_ at the concentration level and called F_0_ at the fluorescence level). An amplification reaction is generally displayed by an amplification curve, while the y-axis represents the fluorescence signal accumulation and the x-axis represents the number of cycles. During the process, the product fluorescence can not rise above the background at the beginning and almost tending to a straight line; as the reaction progresses, the fluorescence accumulates until the product is consumed and the fluorescence ceases to increase [2, 3]. The reason for this process is that, initially, the product quantity is very small, caused a weak fluorescence signal to be detected at baseline phase. The exponential increase of the product starts in cycle 1. It becomes visible when its associated fluorescence can be observed above baseline noise. During the transitional phase products continue to accumulate, but reagents become limiting and the reaction efficiency begins to fall. Until the product is no longer produced, so the reaction reaches to plateau phase [4]. Therefore, the baseline phase, exponential phase, transitional phase, and plateau phase of the amplification curve are generated based on the quantitative relationship between the fluorescence signal accumulation and cycles in Fig. 1A.

In Fig. 1A, the initial fluorescence of the reaction is at the background level with high noise, almost no fluorescence signal can be detected, then the product fluorescence rises above the background in the exponential phase within a few cycles and begins to saturate in the approach to the final plateau phase. Figure 1B shows the locations of relevant parameters determined by C_q_MAN method.

For the relevant parameters of the amplification curve, the amplification process determines a quantitative threshold (called F_q_ in most methods) indicates a detectable fluorescent signal produced by the accumulation of sufficient amplification products which is generally set in the exponential phase. The x-axis of this quantitative threshold corresponds to a cycle called C_q_ in most methods, which is called C_q_MAN in our method.

The amplification efficiency(E) is another important parameter for checking qPCR data analysis. Under ideal conditions, the number of DNA sequences will double in each cycle, the percentage of E-1 is 100% (at this time E is 2) [5]. However, due to factors such as reaction inhibitors, enzyme, primer and probes differences, PCR efficiency rarely reaches 100%. Therefore, E is any number between 1 and 2 [6]. Previously published studies have been suggested that PCR efficiencies mostly range between 65 and 90% [7].

After determining the quantitative cycle, the quantitative threshold, and estimating the amplification efficiency, the kinetics of qPCR exponential phase are described by eq. (1) to indicate the initial target quantity of the reaction.

1 $$ {N}_n={N}_0\times {E}^n $$

in which N_0_ and N_n_, are the initial target amount of DNA and the DNA target amount after n cycles, respectively. F_n_, the fluorescent signal after n cycles and F_0_, the fluorescence signal represents starting amount of the target DNA are the performance of N_n_ and N_0_ at the fluorescence level [2]. Therefore, eq. (1) can be described as eq. (2)

2 $$ {F}_n={F}_0\times {E}^n $$

using the relevant parameters estimated by the curve analysis algorithm method can be expressed as eq. (3)

3 $$ {F}_q={F}_0\times {E}^{C_q} $$

then the observed initial target quantity(F_0_) is calculated.

In the past two decades, the rapid development of qPCR technology has led to the production of multiple protocols, reagents, analytical methods and reporting formats. The original standard-C_q_ method [8, 9] fits a standard curve by preparing multiple sets of replicable experiments of the samples of known concentration, and estimates the concentration of unknown samples from the standard curve. This approach assumes that all standard samples have the same efficiency and is only effective if thresholds are measured from the exponential phase of the PCR reaction, some authors have reported that this assumption may be questionable [10]. Later, an approach proposed by Liu and Saint [11] assumes an efficiency can be obtained by fitting PCR amplification curve with a sigmoid function without preparing standard samples. Since then, the pioneering methods of estimating the target quantity of the initial reaction by calculating the reaction efficiency from the dynamics of a single PCR reaction has been widely used for improvement, and these methods differ in determining the fluorescence baseline, exponential phase, F_q_, C_q_, E to estimate initial target quantities. Baseline estimation is considered a constant baseline in some methods, including the observed minimum fluorescence, the mean value of the three lowest observations, the mean value of a set of fixed cycles [8, 12–14], and the baseline may also be determined by means of a set of dynamically determined baseline phase periods [15, 16] and taking-difference linear regression method [17]. However, the true value of background fluorescence is unknown, and errors in baseline estimation can lead to significant distortion of the results [8, 18, 19]. The difference defined by the exponential phase can easily lead to different results [20]. The residual algorithm estimation with the maximum value of the second derivative as the end point is commonly used [11, 21], or three periods are selected within the midpoint of the fluorescence signal [22]. Estimation of efficiency includes fitting the entire exponential cycle [21, 23], calculating the slope of the points within a certain defined range after linear regression [22, 24], and obtaining the ratio of the threshold fluorescence to the fluorescence value of the previous cycle [25]. F_q_ is generally defined in the exponential phase and then the value of C_q_ is determined, but in some methods, F_q_ and C_q_ are not involved [15, 26, 27]. And the definition process of all parameters may be combined with the fitting of the amplification curve to better obtain [23, 25, 26].

In order to provide reference for further developing and evaluating the qPCR curve analysis method and promoting the research of quantitative fluorescence PCR in gene expression, the new curve analysis method and other methods were evaluated on the biomarker dataset and 94-replicates-4-dilutions set in this paper from the aspects of expression level and statistical significance. The goal of this paper is to make our new method a comparison of other methods, at the same time provide users with an alternative curve analysis scheme. In order to evaluate the new method, some evaluation performance indicators were proposed.

## Methods

### qPCR dataset

#### Biomarker dataset

Data comes from a previously published study [28] that developed and validated the expression profile of a 59-mRNA gene to improve prognosis in children with neuroblastoma. This dataset measured 59 biomarkers and 5 reference genes in a sample maximization experimental design, using the LightCycler480 SYBR Green Master (Roche) in a 384-well plate with 8 μl reaction. These genes have been reported in at least two independent studies as prognostic genes for neuroblastoma. Three hundred sixty-six cDNA samples from the primary tumor biopsy and a 5-point 10-fold serial dilution series based on an external oligonucleotide standards (from 150,000 to 15 copies, *n* = 3), and no template control (NTC, *n* = 3) are included in each plate [28, 29]. This dataset will be referred to as ‘biomarker dataset’ in this study. Since there was no obvious specificity of 63 genes in this dataset, 20 of them (AHCY,AKR1C1,ARHGEF7,BIRC5,CAMTA1,CAMTA2,CD44,CDCA5,CDH5,CDKN3,CLSTN1,CPSG3,DDC,ECEL1,ELAVL4,EPB41L3,EPHA5,EPN2,FYN,HIVEP2) were randomly selected and then 300 (5 × 3 × 20) amplification curve data of 20 genes with concentration of 150,000, 15,000, 1500, 150, 15(3 replicated experiments for each group) were used for subsequent analysis.

#### 94-replicates-4-dilutions set

This data set created a dilution series consisting of four 10-fold serial dilution points from 15,000 to 15 molecules, using 10 ng / μl yeast tRNA as a carrier (Roche) and created NTC samples of the same dilution. qPCR was done on a CFX 384 instrument (Bio-Rad). QPCR was performed on a CFX 384 instrument (Bio-Rad) using a 96-well pipetting robot (Tecan Freedom Evo 150). Amplification reactions were performed in 8 μl samples containing 0.4 μl forward and 0.4 μl reverse primer (5 μM each), 0.2 μl nuclease-free water, 4 μl iQ SYBR Green Supermix (Bio-Rad) and 3 μl of standard oligonucleotide. In 384-well plates (Hard-Shell 384-well microplate and Microseal B clear using an adhesive seal (Bio-Rad)), for each of the 4 dilution points, a total of 94 replicate reactions were distributed. In addition, the NTC reaction was repeated 8 times [28]. This dataset will be referred to as ‘94-replicates-4-dilutions set’. And 44 (4 × 11) amplification curves of the MYCN gene with a diluted concentration of 15, 150, 1500,15,000(11 replicated experiments for each group) were used for subsequent analysis.

### qPCR curve analysis method

#### Previously published curve analysis method

We provide general descriptions of the 7 methods previously published. In this study, these methods will be referred to with their preferred abbreviations LinRegPCR, DART, FPLM, FPK-PCR, 5PSM, PCR-Miner and Cy0. The LinRegPCR program [16] starts with import of raw fluorescence data. A constant baseline fluorescence is determined per reaction with an iterative algorithm that aims at the longest set of data points on a straight line going down from the second derivative maximum cycle. After subtraction of the baseline fluorescence, LinRegPCR sets a window-of-linearity (W-o-L) that includes 4 points in the exponential phase of each sample and calculates the individual PCR efficiency from the slope of the regression line through these points. For each amplicon group, a quantification threshold F_q_ is set at 1 cycle below the top border of the W-o-L and the C_q_ is determined for each reaction. DART [22] constructs a model based on the maximum fluorescence value (R_max_) and the baseline fluorescence noise (R_noise_) to determine a central point M, and fits the cycle within a 10-fold range around M to estimate E, F_q_, C_q_ obtain by 10-fold the standard deviation of 1–10 cycles. FPLM [21] uses four-parameter logistic model to fit the fluorescence curve and estimate the exponential phase, and the same as DART in determining F_q_, C_q_. The bilinear model and the six-parameter logistic model are used in the FPK-PCR [26] to estimate the E and initial target quantity without determining fluorescence threshold.5PSM [25] uses the ratio of the fluorescence value at the second derivative maximum (SDM) after fitting the curve with the five-parameter model to the fluorescence value of the previous cycle as the amplification efficiency and the cycle of SDM is used as the C_q_. The principle of PCR-Miner [30] is based on the four-parameter logistic model to fit the raw fluorescence data as a function of PCR cycles to identify the exponential phase of the reaction. The method chooses the first positive second derivative maximum from the logistic model to calculate the dynamic fluorescence threshold and corresponding C_q_. A three-parameter simple exponent model is fitted to this exponential phase using an iterative non-linear regression algorithm to compute the individual efficiency. Cy0 [31] obtains the intersection point (Cy0) between the abscissa axis of the curve inflection point and the tangent line based on the nonlinear regression of the Richards equation to the fluorescence value. The efficiency is estimated by the parameters in the post-fitting equation, and then the initial target quantity is obtained.

#### C_q_MAN method

C_q_MAN (C_q_ Management And Analysis System) is an adaptive analysis system that summarizes the methods and experiences of previous methods and provides a robust, objective, and noise-resistant method for quantification of qPCR results. Since researches have shown that smoothing can at best lead to erroneous accuracy of results, and usually also bias the results [32], the improved adaptive Savitzky-Golay filter in the C_q_MAN system is only used for visual display of data. The detailed process is shown in Additional file 1. The C_q_MAN method has been implemented in the system. We provide the URL of the system (http://122.193.29.190:9913/xMAN/en-us/index), and readers can reproduce our experimental results by combining with Additional files 1 and 2.

C_q_MAN method relies on the modified gompertz model, is fitted to the raw fluorescence data by means of a non-linear fitting routine the Levenberg-Marquardt algorithm that minimizes the residual sum-of-squares to obtain parameters baseline fluorescence (y_0_) and maximum fluorescence (y_max_), exp. is the natural logarithm base, Ln is the natural logarithm, x is the actual cycle number, b and x_0_ determine the shape of each model.

4 $$ y={y}_0+\left({y}_{max}-{y}_0\right){\lambda}^{{-\mathit{\exp}}^{-\left(x-{x}_0\right)/b}} $$

5 $$ {F}_{SDM}= bLn\left(\left(\left(\sqrt{5}-3\right){\lambda}^{\frac{X_0}{b}}\right)/2\right) $$

**Fig. 2:**
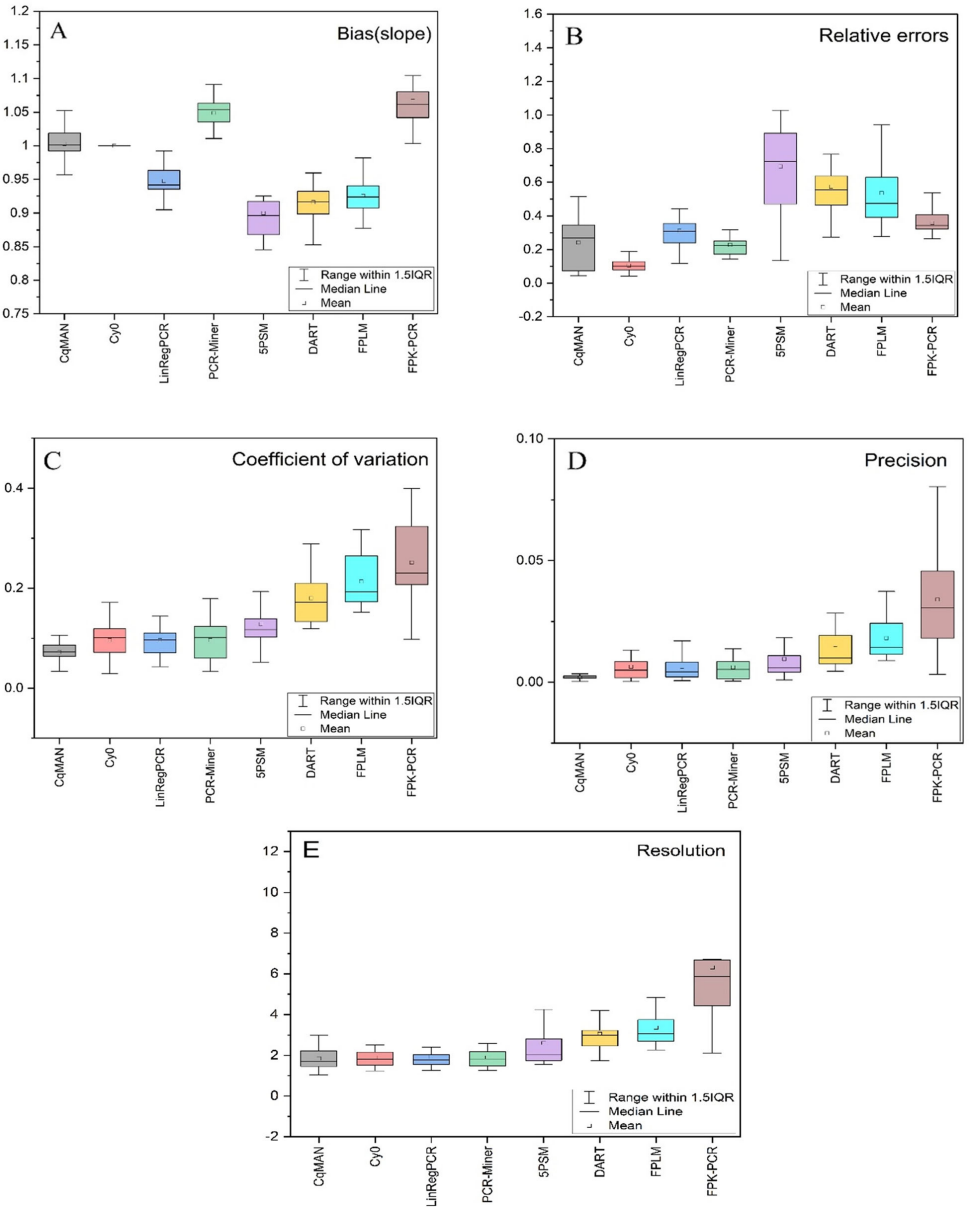
Performance indicators per method. (OriginPro 2020b https://www.originlab.com/)

The maximum value of the second derivative are obtained by fitting the second derivative of the gompertz curve to estimate the end of the exponential phase (eq. (5)). x_SDM_ is the cycle at the maximum of the second derivative (SDM) which is applied as the end point of the exponential phase and the fluorescence value corresponding to this cycle is F_SDM_ in C_q_MAN method. Take the intermediate value of y_0_ and F_SDM_ as the “midpoint” F_q_ (eq. (6)), then substitute this value into eq. (4) to obtain the quantitative cycle (C_q_MAN) (see Fig. 2B).

6 $$ {F}_q=\left({y}_0+{F}_{SDM}\right)/2 $$

For efficiency estimation, a three-parameter simple exponent model is fitted to this exponential phase (from C_q_MAN to x_SDM_) using an non-linear regression algorithm to estimate the single reaction’s individual efficiency in eq. (7). The individual efficiency of multiple reactions of the same gene is averaged, then the observed target quantity (F_0_) can be calculated by eq. (8).

7 $$ {F}_n={F}_q+\alpha \times {E}^n $$

8 $$ {F}_0=1/{E_{\boldsymbol{mean}}}^{C\mathrm{q} MAN} $$

The logistic model used in Cy0, PCR Miner are generally susceptible to the influence of the number of amplified data in the plateau phase, resulting in inaccurate fitting [33]. At the same time, 5PSM adds a parameter to the logistic model to maintain the symmetry of the s-shaped curve structure, which will affect the calculation of parameters such as the maximum of the second derivative, resulting in larger errors. The gompertz model in C_q_MAN is not easily affected by the data in the plateau phase, and it fits well in all the phase. At the same time, this method can ensure that the C_q_MAN value is within the exponential phase without judging the starting point of the phase (in the first 2–3 cycles of the cycle where the SDM is located). It does not rely on baseline estimation of the noise larger phase of the fluorescence signal, and avoids the problem of deviation caused by the assumption in the DART and FPLM method that a constant baseline can be determined from the baseline phase. By using nonlinear regression fitting to estimate the average efficiency of all reactions of each gene, C_q_MAN method further averaged the amplification reaction noise between each gene, more effectively resisting the noise while reducing the estimation error [31]. However, the shortcoming is that this method is prone to error under the influence of dynamic outliers (inhibition), in which aspect FPK-PCR performs better.

## Results

### Performance indicators

To eliminate the different measurement scales used by the analytical method based on concentration levels and fluorescence levels [34], we divided the data of all concentrations by the highest concentration data and all fluorescence data by the average value of the maximum observed target quantity (F_0_), so that the average value of the maximum concentration and the maximum observed target quantity is 1. This process is called normalization. Then data sets were used to establish 6 performance indicators to measure the degree of compliance between the observed initial target quantity (F_0_) calculated by the algorithm and the true value from different angles. Among them, the bias and relative error are used to compare the difference between the observed initial target quantity and the true value; coefficient of variation and precision are used to compare the difference between the observed initial target quantity (F_0_) of the same group. The smaller the difference, the more reliable the method. Performance indicators as follows.

(1) Bias. The ratio between the average of the observed initial target quantity F_0_ corresponding to the highest and lowest concentrations is calculated. In biomarker, the expected value of this ratio is 10,000 (because the ratio of the concentration of 150,000 and 15 is 10,000), and in 94-replicates-4-dilutions set, the expected value of this ratio is 0.001 (because the ratio of the diluted concentration of 15 and 15,000 is 0.001) and any value deviating from 10,000 or 0.001 is expressed as a bias. The log-transformed (base 10) between the true value and the initial target quantity F_0_. After the data is normalized, the linear regression analysis makes the log (F_0_) and log (NC) (NC, normalized concentration) slopes of the unbiased method 1 and any slope deviates from the value of 1 also expressed as a bias.

(2) Relative error (RE).

9 $$ RE=\frac{F_0- NC}{NC} $$

RE is the deviation after F_0_ and NC are normalized to the same measurement scale.

(3) Coefficient of variation (CV).

10 $$ CV=\frac{SD_{\boldsymbol{group}}}{\mu_{\boldsymbol{group}}}\times 100\% $$

CV represents the ratio of the standard deviation (SD) to the average value(μ) of the same group (replicated experiments) of observed initial target quantity (F_0_).

(4) Precision. Precision represents the within-triplicate variance of the observed initial target quantity (F_0_) in the same group.

(5) Resolution. A linear regression analysis of log (true) on log(F_0_) was performed and the 95% CI around the regression line was constructed. The width of this interval was converted into a fold deviation from the regression line and the geometric mean for the 5 groups was calculated as a measure of resolution.

### Indicator evaluation

In the supplementary information, the original amplification experiment data of the two data sets used in this study were obtained from Reference [28] after being processed into the readable format of the C_q_MAN system. We imported the data of these two data setsinto the C_q_MAN system to obtain the F_0_, C_q_, and E calculated by the C_q_MAN, integrated the results with the three parameter values of the other 7 methods provided in reference [28] (see biomarker_performance _indicators and 94_replicates_4_dilutions_set_results). In the bias_and_deviat_from_regres of biomarker_performance_indicators, the process of C_q_MAN calculating 4 performance indicators is shown and it is the same as the calculation process of other 7 methods. Therefore, the calculation process of the other 7 methods is no longer provided. The performance indicators’ calculation results of the 8 methods are provided in biomarker_analysis_dilutoin_series in and 94_replicates_4_dilutions_set_results.

**Fig. 3:**
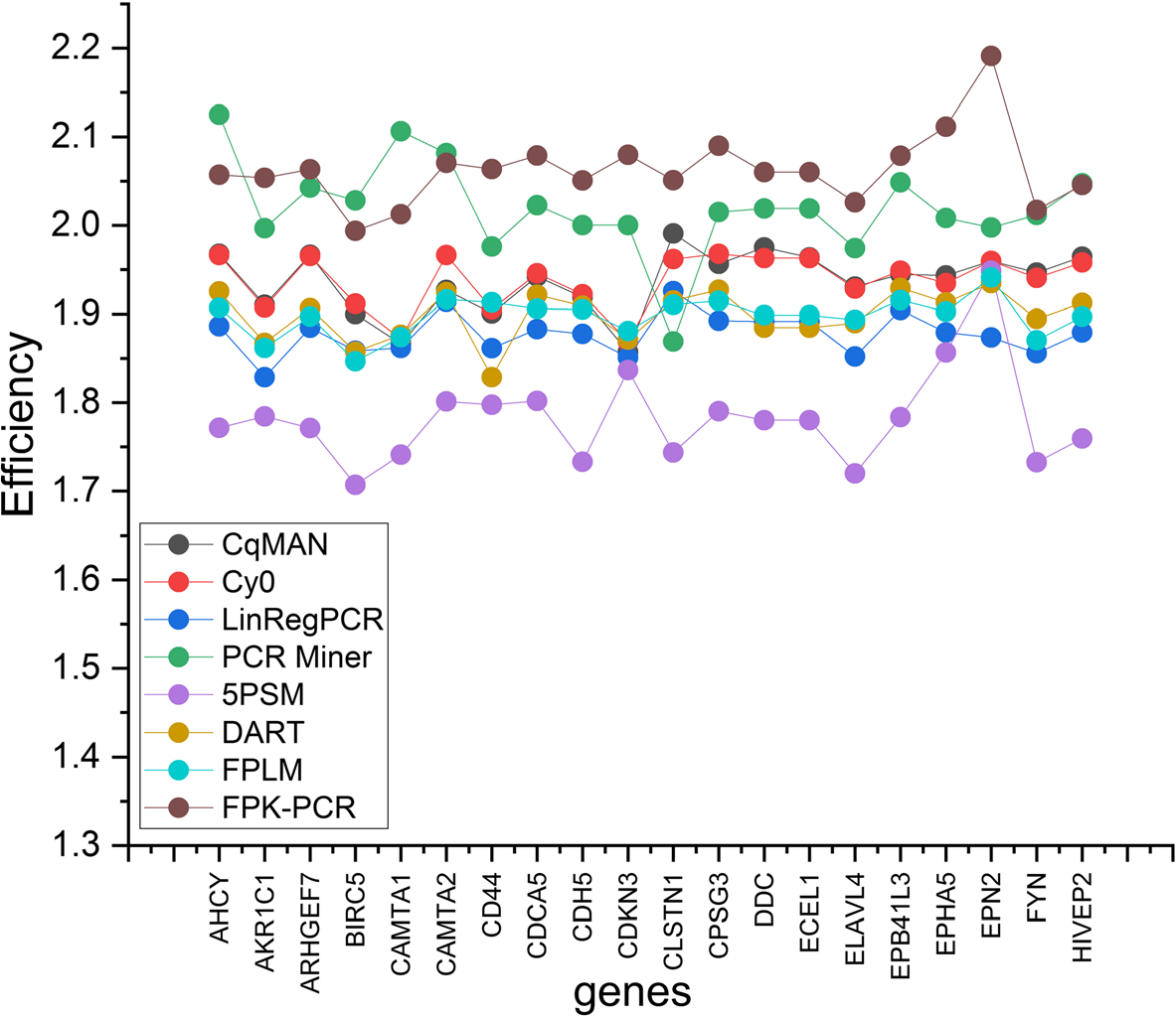
PCR efficiency per gene. (OriginPro 2020b https://www.originlab.com/)

Except that the efficiency analysis results of the other 7 methods (see Fig. 3) directly used the data provided in reference [28] in the subsequent performance indicator analysis, the analysis results of other performance indicators are all reanalysis results.

### Biomarker dataset analysis

The performance indicator values determined from the concentration series included in the measurement of the 20 genes are summarized in box-and-whisker plots. The boxes range from the 25th to the 75th percentile and are divided by the median; the whiskers are set at the 5th and 95th percentile (A) Bias in the slope level, which is based on the degree of deviation from 1.(B) The box-and-whisker plot of relative errors shows the difference between the observed initial target quantity and the true value.(C) Coefficient of variation is an objective indicator of the effects of measurement scales and dimensions that eliminate fluorescence levels and concentration levels.(D) Precision is determined as the within-triplicate variance and should have the same, low, value in all methods.(E) Resolution defined as the fold-chance that would result in the detection of a difference at a 5% significance level.

The mean value of the efficiencies of each gene per method.

(1) Bias. We expect the ratio between the observed initial target quantity and the true value to be 10,000 or 0.001 in two different datasets. After the data is normalized, the linear regression analysis makes the log (F_0_) and log (SQ) slopes of the unbiased method 1, which will be unbiased. Cy0 has an advantage in the deviation index because the method calculate the efficiency value based on the slope of the relationship between Cy0 and log (input), and then use this efficiency value and the Cy0 value to calculate F_0_. Therefore, Cy0 is unbiased and are the result of circular reasoning, but this also ensures that the observed initial target quantity F_0_ is more accurate. Other methods are positively or negatively biased, and the observed values deviate significantly from the true values in Fig. 2A. Among them, C_q_MAN performs better in the bias, with an average deviation of 2469.0003(for 10,000) and 0.0182(for 0.001).

(2) Relative error. The relative error was originally used to compare the difference between the measured value and the true value, and the degree of confidence in the response measurement. Here we can use the relative error response to calculate the difference between the observed value and the true value, reflecting the credibility of the algorithm. More intuitive response measurement accuracy than absolute error. We use relative error as one of the indicators to determine the difference between the observed initial target quantity F_0_ and the true value. Cy0 performed best, average relative error was 0.1050. The average relative error of the rank after the second PCR-Miner was 0.2287, C_q_MAN was 0.2416, and the highest 5PSM was as high as 0.6939 in Table 1 and Fig. 2B.

**Table 1:** Analysis of the average of 20 genes in 4 indicators per method

Methods	Bias(10000)	Bias(1)	Relative error	Coefficient of variation	Precision	Resolution
**C**_**q**_**MAN**	2469.0003	0.0182	0.2416	7.20%	0.0020	1.9222
**Cy0**	2594.5631	0.0000	0.1050	9.62%	0.0064	1.9213
**LinRegPCR**	3891.3018	0.0523	0.3117	9.64%	0.0064	1.9051
**PCR Miner**	6167.5939	0.0561	0.2287	9.63%	0.0062	1.9156
**5PSM**	6041.4519	0.0999	0.6939	12.89%	0.0096	3.0166
**DART**	5233.9256	0.0840	0.5727	17.98%	0.0140	2.8630
**FPLM**	4814.3920	0.0745	0.5357	21.34%	0.0182	3.1956
**FPK-PCR**	8536.6094	0.0669	0.3592	25.12%	0.0339	5.8404

(3) Coefficient of variation. The coefficient of variation reflects the degree of dispersion of the data, and at the same time overcomes the effects of large differences in measurement scales or different data sizes. We use the coefficient of variation coefficient to calculate the degree of dispersion of the observed initial target quantities of the three groups at each concentration, and average the five groups of coefficients of variation. The smaller the coefficient of variation, the lower the degree of dispersion. Result showed that C_q_MAN showed the best performance of 7.20%, Cy0, LinRegPCR, PCR-Miner also stabilized at about 9.60%, and FPK-PCR’s coefficient of variation was as high as 25.12% in Table 1 and Fig. 2C.

(4) Precision. The five concentration sequences were measured three times and the fluorescence data were analyzed. Therefore, the variance of each set of 3 measurements should be small, reflecting only random changes in laboratory procedures and fluorescence measurements, and such changes should always be the same. The resulting three internal variances can be considered as a measure of the accuracy of the analytical method. C_q_MAN, 5PSM, Cy0, LinRegPCR have lower variability in Fig. 2D.

(5) Resolution. Data points outside the 95% CI of the regression line fitted to the concentration sequence after linear regression will be judged to be significantly different from the true value and expressed in resolution. LinRegPCR has the lowest resolution; lower is better. Cy0, PCR-Miner and C_q_MAN also perform well in Fig. 2E. With these 4 methods, the observed 2-fold difference is significant for approximately 85% of genes. For 5PSM, DART, FPLM, the resolution lies between the 2 and 3-fold-difference. In FPK-PCR, 40% of genes are over 5-fold-difference.

(6) Efficiency. The range of differences in efficiency values for each method indicates that this variability is the sum of the difference in efficiency between genes and the difference in estimation methods. Therefore, the difference between the methods cannot be explained. Except that DART and FPLM share a method of finding E, other methods get different median values of E. FPK-PCR and PCR-Miner have a large number of efficiency values above 2, which is obviously too high and the median value of C_q_MAN, Cy0, LinRegPCR, 5PSM is between 1.7 and 1.9. We calculated the standard deviation of the amplification efficiency of the 20 genes, in which LinRegPCR, DART, FPLM calculated E value is relatively stable in Fig. 3.

### 94-replicates-4-dilutions set analysis

The highest dilute concentration is set to 1, the y-axis is set to log (dilution) (base 10).

**Fig. 4:**
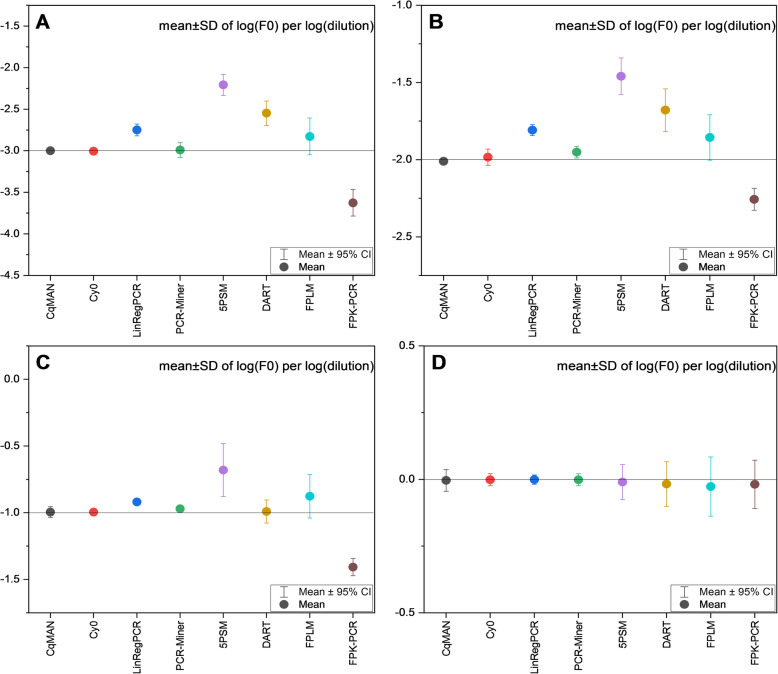
Mean observed F_0_ value per concentration and method. (OriginPro 2020b https://www.originlab.com/)

(1) Target quantity. For data with dilute concentrations of 15,000, 1500, 150, and 15, respectively, the observed target quantity should be as close as possible to the expected value −3, −2, −1, 0 obtained after calculating the log (F_0_) (base 10) in Fig. 4. The systematic negative or positive deviation of each analysis method is shown by the deviation of the average F_0_ from the expected value (Fig. 4: horizontal line). C_q_MAN, Cy0, PCR-Miner and LinRegPCR have the least bias. DART and FPLM show a higher bias, 5PSM displays a strong overestimation whereas FPK-PCR shows a strong underestimation of F_0_ values.

(2) Bias, RE, CV, precision, resolution and E. C_q_MAN and Cy0 keep lower variance in bias. C_q_MAN perform best in RE, CV and precision. C_q_MAN, Cy0, LinRegPCR and PCR-Miner does not vary much between the values in CV and precision. LinRegPCR has the lowest resolution, the average resolution of Cy0, PCR-Miner and C_q_MAN is around 2-fold. Table 2 clearly illustrate the differences in 6 indicators of 8 methods and the average PCR efficiency of these methods is provided. The efficiency of Cy0 was not provided in the previously published data analysis.

**Table 2:** Analysis of MYCN gene in 4 indicators and the mean of PCR efficiency per method

Methods	Bias(0.001)	Bias(1)	Relative error	Coefficient of variation	Precision	Resolution	^**−**^E
**C**_**q**_**MAN**	0.0000	0.0000	0.0873	10.86%	0.0025	2.0132	1.8300
**Cy0**	0.0000	0.0000	0.0935	10.42%	0.0027	2.1344	NA
**LinRegPCR**	0.0008	0.0869	0.4126	11.32%	0.0039	1.5326	1.8690
**PCR-Miner**	0.0001	0.0054	0.1434	12.39%	0.0057	2.0524	1.9905
**5PSM**	0.0057	0.2633	2.5536	41.40%	0.0409	7.0584	1.7462
**DART**	0.0022	0.1723	1.0169	39.86%	0.0308	7.1726	1.9047
**FPLM**	0.0008	0.0617	0.6724	50.71%	0.0609	7.4493	1.9844
**FPK-PCR**	0.0007	0.1672	0.5012	29.55%	0.0236	7.8181	2.3011

## Discussions

For each of the evaluation indexes of the concentration sequence analysis of each gene, the rank synthesis method was used, and the Friedman test determined that these methods were not significantly different and comparable. Table 3 shows the results of each gene and method. The lower average rank indicates that the method which estimates the initial target quantity is closer to the true value in the performance evaluation of the four indicators we selected.

**Table 3:** Analysis of performance parameters per method in biomarker dataset (left) and 94-replicates-4-dilutions set (right). For each method, the mean rank is given for each of the performance indicators bias, RE, CV, precision and resolution. The methods are sorted based on the average of these ranks

Methods	Bias(10,000/0.001)	Bias(1)	Relative error	Coefficient of variation	Precision	Resolution	rank
**C**_**q**_**MAN**	2/2	1/1	3/1	1/2	1/1	4/2	2.08/1.58
**Cy0**	1/1	1/1	1/2	2/1	4/2	3/1	2.08/1.92
**LinRegPCR**	3/6	2/4	4/4	4/3	3/3	1/4	3.00/3.67
**PCR-Miner**	7/3	3/2	2/3	3/4	2/4	2/3	3.33/3.33
**5PSM**	6/8	7/7	8/8	5/7	5/7	6/5	6.33/7.67
**DART**	5/7	6/6	7/7	6/6	6/6	5/6	6.00/6.67
**FPLM**	4/5	5/3	6/6	7/8	7/8	7/7	6.17/6.17
**FPK-PCR**	8/4	4/5	5/5	8/5	8/5	8/8	7.00/5.00

In the average rank sorting of 20 genes in the biomarker data set, the lowest rank average of C_q_MAN and Cy0 are 2.08. The rank averages of the 5PSM, DART, FPLM, and FPK-PCR are all above 6, and the overall performance of F_0_ estimation is lower in Table 3. For the 94-replicates-4-dilutions set, the performance of C_q_MAN is 1.58, the average rank of Cy0 is 1.92, and the performance of LinRegPCR and PCR-Miner are also good; the rank average of 5PSM, DART, FPLM, and FPK-PCR is much higher.

## Conclusions

Based on PCR kinetics and exponential model simulations, this study combines the real-time quantitative PCR curve analysis method proposed by the predecessors, and proposes a reliable gene expression level quantification method, C_q_MAN. To prove the reliability of the method, two data sets from different instruments, different PCR mixtures, and a testable hypothesis were used to evaluate the performance of multiple qPCR curve analysis methods. The fluorescence data of the other 7 methods in the performance analysis process were taken from a previously published research by Ruijter et al. in 2013 [28]. Since the supplemental information from this research provided an excel template for calculating bias and precision, we can directly import the amplification curve data from two data sets analyzed by the C_q_MAN system into the excel template to obtain the calculated values of the two indicators. The relative error and coefficient of variation are the two statistical indicators proposed by the author of this study for evaluation and analysis. Therefore, due to the difference in indicator settings and the difference in data sets selection, our analysis results are different from the results previously published by Ruijter et.al.

The limitation of this study is that two datasets have limited evaluation of the general applicability of the C_q_MAN method, so future researches should include more instances and more verification indicators to better verify the robustness and representativeness of the method. However, it is undeniable that the analysis templates, datasets, and analysis results (see supporting information) in this research will definitely help further evaluation of research and make the results comparable with our results.

The aim of this study is not to promote a particular curve analysis method with the best overall performance, because the choice of methods by the experimenters may depend on the different research goals of experimental instruments, reagents, protocols, etc. It is our intention to help users choose the ideal method for their own studies and developers to modify and improve their methods [35].

## Supplementary Information

**Additional file 1.**

**Additional file 2.**

## Data Availability

http://122.193.29.190:9913/xMAN/en-us/index

## Abbreviations

RT-qPCR: Reverse transcription quantitative polymerase chain reaction
C_q_MAN: C_q_ management and analysis system
RE: Relative error
CV: Coefficient of variation
E: Efficiency
