# Supplementary material for: Evaluation validation of a qPCR curve analysis method and conventional approaches
Source: BMC Genomics. 2021 Nov 16;22(Suppl 5):680. doi: 10.1186/s12864-021-07986-4 (PMC8596907; doi:10.1186/s12864-021-07986-4)
Supplement: Supplementary file 1 — Additional file 1. [file 12864_2021_7986_MOESM1_ESM.pdf]

Principle of adaptive Savitzky-Golay filter in C_qMAN System amplification curve visualization processing

Savitzky-Golay is a filter based on the local polynomial least squares convolution algorithm in the time domain. It reduces noise while better maintaining the shape of the signal, and retains the distribution characteristics of the data points such as relative maximum, minimum, and width .

C_qMAN system sets the width n of the filter window to 5 (n=2m+1), slide in steps of 5 and fits fluorescence values y_m, y_{-m+1}, ..., y_0, y_1, ..., y_{m-1}, y_m within the window width, using k-1 degree polynomial(equation (1)) for fitting

$$y = a_0 + a_1 x + a_2 x^2 + \cdots + a_{(k-1)} x^{(k-1)} \tag{1}$$

Therefore, there are n equations to constitute the k-ary linear in equation (2). To make the equations have a solution, n should be greater than or equal to k, generally choose n>k, set to 4 in the system, and determine the fitting parameters a_0, a_1, …, a_{k-1} by least squares.

$$\begin{bmatrix} y_{-m} \\ y_{-m+1} \\ \vdots \\ y_m \end{bmatrix} = \begin{bmatrix} 1 & -m & \cdots & (-m)^{k-1} \\ 1 & -m+1 & \cdots & (-m+1)^{k-1} \\ \vdots & \vdots & \vdots & \vdots \\ 1 & m & \cdots & m^{k-1} \end{bmatrix} \begin{bmatrix} a_0 \\ a_1 \\ \vdots \\ a_{k-1} \end{bmatrix} + \begin{bmatrix} e_{-m} \\ e_{-m+1} \\ \vdots \\ e_m \end{bmatrix} \tag{2}$$

The matrix form of equation (2) is expressed as

$$\boldsymbol{Y}_{(2m+1)\times 1} = \boldsymbol{X}_{(2m+1)\times k} \cdot \boldsymbol{A}_{k+1} + \boldsymbol{E}_{(2m+1)\times 1} \tag{3}$$

The least squares solution A’ of A is

$$\boldsymbol{A}' = (\boldsymbol{X}^T \cdot \boldsymbol{X})^{-1} \cdot \boldsymbol{X}^T \cdot \boldsymbol{Y} \tag{4}$$

And the filtered fluorescence value matrix Y' is

$$\boldsymbol{Y'} = \boldsymbol{X} \cdot \boldsymbol{A'} = (\boldsymbol{X}^T \cdot \boldsymbol{X})^{-1} \cdot \boldsymbol{X}^T \cdot \boldsymbol{Y} \tag{5}$$

After that, the improved adaptive Savitzky-Golay filter makes the reconstructed amplification curve obtain the best filtering effect with the smallest fitting index(P_k) through an iterative process.

(a) Before the iteration, the first Savitzky-Golay smoothing process is performed to obtain the total trend line (Y^t) of the curve.

(b) The k-th iteration operation replaces the value of the trend line with a value lower than the total trend line to generate a new sequence (Y^k).

(c) Use the iteration result with the smallest fitting index ($P_{k)}$ after the k-th iteration as the final filtering result.

$$P_k = \sum_{i=1}^{N} [| Y_i^{k+1} - Y_i^0 | \times w_i] \tag{6}$$

In equation (6), Y_i^0 and Y_i^{k+1} represent the i-th fluorescence value without iteration and the i-th fluorescence value after k-th iteration, w_i represents the weight of the i-th fluorescence value. The method for determining the weight is as follows:

(a)The weight of all fluorescence values at the first smoothing is 1.

(b)When iterating, w_i is obtained from equation (7).

$$w_i = \{ \begin{matrix} 1, & Y_i^0 \geq Y_i^t \\ 1 - d_i / d_{max}, & Y_i^0 \leq Y_i^t \end{matrix} \tag{7}$$

In equation (7), $d_i = |Y_i^0 - Y_i^t|$ represents the distance between the uniterated i-th fluorescence value and the total trend line, and d_{max} represents the distance between the largest uniterated i-th fluorescence value and the total trend line.
